# The *ZC3HC1* rs11556924 polymorphism is associated with increased carotid intima-media thickness in patients with rheumatoid arthritis

**DOI:** 10.1186/ar4335

**Published:** 2013-10-11

**Authors:** Raquel López-Mejías, Fernanda Genre, Mercedes García-Bermúdez, Alfonso Corrales, Carlos González-Juanatey, Javier Llorca, José A Miranda-Filloy, Javier Rueda-Gotor, Ricardo Blanco, Santos Castañeda, Javier Martín, Miguel A González-Gay

**Affiliations:** 1 Epidemiology, Genetics and Atherosclerosis Research Group on Systemic Inflammatory Diseases, Rheumatology Division, IFIMAV, Avenida de Valdecilla, s/n, Santander 39008, Spain; 2 Instituto de Parasitología y Biomedicina López-Neyra, IPBLN-CSIC, Granada, Spain; 3 Cardiology Division, Hospital Lucus Augusti, Lugo, Spain; 4 Department of Epidemiology and Computational Biology, School of Medicine, University of Cantabria, and CIBER Epidemiología y Salud Pública (CIBERESP), IFIMAV, Santander, Spain; 5 Division of Rheumatology, Hospital Lucus Augusti, Lugo, Spain; 6 Rheumatology Department, Hospital Universitario la Princesa, IIS-Princesa, Madrid, Spain

## Abstract

**Introduction:**

Rheumatoid arthritis (RA) is a complex polygenic disease associated with chronic inflammation, accelerated atherosclerosis and increased cardiovascular (CV) mortality. A recent meta-analysis has described the *ZC3HC1* rs11556924 polymorphism as one of the most important signals associated with coronary artery disease (CAD) in non-rheumatic Caucasian individuals. In this study we evaluated the potential association of this gene polymorphism with subclinical atherosclerosis assessed by the evaluation of carotid intima-media thickness (cIMT) in RA patients.

**Methods:**

This study included 502 RA patients from Northern Spain. The *ZC3HC1* rs11556924 polymorphism was genotyped with TaqMan single-nucleotide polymorphism (SNP) genotyping assays (C__31283062_10) in a 7900HT real-time polymerase chain reaction (PCR) system. cIMT was also assessed in these patients by carotid ultrasonography (US) technology.

**Results:**

RA patients carrying the TT genotype had significantly higher cIMT values than those homozygous for the CC genotype (mean ± standard deviation (SD): 0.76 ± 0.18 mm and mean ± SD: 0.71 ± 0.16 mm respectively; *P* = 0.03) even after adjusting the results for sex, age at the time of US study, follow-up time and traditional CV risk factors (*P* = 0.04) evidencing that the effect conferred by *ZC3HC1* rs11556924 polymorphism is independent of the traditional CV risk factors.

**Conclusion:**

Our results indicate that *ZC3HC1* rs11556924 polymorphism is associated with subclinical atherosclerosis in RA.

## Introduction

 Rheumatoid arthritis (RA) is a complex inflammatory disease associated with increased risk of cardiovascular (CV) disease and CV mortality that is the result of accelerated atherosclerosis [1,2]. Because of that, adequate stratification of the CV risk has special relevance in patients with RA. Besides traditional CV risk factors and chronic inflammation [3], recent studies have also highlighted the implication of genetic factors and the influence of several gene polymorphisms in the susceptibility to and/or in the risk of accelerated atherosclerosis of patients with RA [4]. Since CV disease is the most common cause of premature mortality in RA, an important step forward might be to identify high-CV risk RA patients who would benefit from active therapy to prevent the development of CV complications.

Subclinical atherosclerosis has been observed in patients with RA [5], even in those without traditional CV risk factors [5]. Several validated noninvasive imaging techniques are currently available to determine subclinical atherosclerosis in RA. They can offer a unique opportunity to study the relation of surrogate markers to the development of atherosclerosis [6]. Among them, by the assessment of carotid intima-media thickness (cIMT), carotid ultrasonography (US) has become an affordable efficient technique to measure the presence of subclinical atherosclerosis. A meta-analysis encompassing several population based studies confirmed an increased cIMT in RA patients when compared with the general population [7]. Interestingly, as observed in the general population, abnormally high values of cIMT (greater than 0.90 mm) have been found to predict the development of CV events in patients with RA after five years of follow-up [8].

Recently, a meta-analysis of 14 genome-wide association studies of coronary artery disease (CAD) performed in non-rheumatic Caucasian individuals has identified 13 novel loci harboring one or more polymorphisms that were associated with this pathology and confirmed the association of 10 of 12 previously reported CAD loci [9]. With respect to this, the genetic variant rs11556924 (C > T) that is located at 7q32.2 and encodes a non-synonymous change (R363H) in the *ZC3HC1* (*zinc finger, C3HC-type containing 1*) gene, seems to be one of the most significant signals associated with CAD in non-rheumatic Caucasian individuals [9].

Taking all these considerations into account, in the present study we aimed to determine, for the first time, the potential association between the *ZC3HC1* rs11556924 polymorphism (as a marker of CV disease) and subclinical atherosclerosis manifested by the increase of cIMT in patients with RA.

## Methods

### Patients and study protocol

A total of 502 Spanish patients with RA from Northern Spain were included in the present study. Blood samples were obtained from patients recruited from Hospital Lucus Augusti (Lugo) and Hospital Marqués de Valdecilla (Santander). Ethics Committees of Cantabria for Hospital Universitario Marqués de Valdecilla in Santander and Galicia for Hospital Lucus Augusti in Lugo approved the work. Patients gave the necessary written consent, including consent to participate in the study and consent to publish the results.

All the patients fulfilled the 1987 American College of Rheumatology (ACR) and also the 2010 classification criteria for RA [10,11]. In all the cases, patients were assessed for the *ZC3HC1* rs11556924 polymorphism.

Information on the main demographic and clinical characteristics of the patients enrolled in the study, CV risk factors and CV events of these patients is shown in Table 1. Definitions of CV events (ischemic heart disease, heart failure, cerebrovascular accident or peripheral arteriopathy) and definitions of traditional CV risk factors were established as previously described [8,12].

**Table 1:** Demographic and clinical characteristics of the Spanish patients with RA included in the study

**Clinical feature**	**% (n/N)**
Patients	502
**Main characteristics**	
Age at the time of disease onset (years, mean ± SD)	50.8 ± 14.5
Follow-up (years, mean ± SD)	9.5 ± 8.1
Percentage of women	77.7
Rheumatoid factor positive^a^	60.6 (297/490)
Anti-CCP antibodies positive	51.9 (227/437)
Shared epitope positive	62.1 (179/288)
Extra-articular manifestations^b^	21 (107/502)
**Cardiovascular risk factors**	
Hypertension	22.7 (110/484)
Diabetes mellitus	6.6 (32/484)
Dyslipidemia	15.1 (73/484)
Obesity	9.7 (47/484)
Smoking habit	27.9 (135/484)
**Patients with cardiovascular events**	10.7 (54/502)
Ischemic heart disease	4.6 (23/502)
Heart failure	1.9 (10/502)
Cerebrovascular accident	3.8 (19/502)
Peripheral arteriopathy	1.2 (6/502)

^a^At least two determinations were required; ^b^extra-articular manifestations of the disease (if RA patients experienced at least one of the following manifestations: nodular disease, Felty’s syndrome, pulmonary fibrosis, rheumatoid vasculitis, or secondary Sjögren’s syndrome) [12]. Anti-CCP antibodies, anti-cyclic citrullinated peptide antibodies; RA, rheumatoid arthritis; SD, standard deviation.

### Genotyping

DNA from patients was obtained from peripheral blood using standard methods.

The *ZC3HC1* rs11556924 polymorphism was genotyped with TaqMan predesigned single-nucleotide polymorphism (SNP) genotyping assays (C__31283062_10) in a 7900 HT Real-Time polymerase chain reaction (PCR) system, according to the conditions recommended by the manufacturer (Applied Biosystems, Foster City, CA, USA). Negative controls and duplicate samples were included to check the accuracy of genotyping.

### Carotid ultrasonography examination

Patients from Santander were assessed using a commercially available scanner, Mylab 70, Esaote (Genoa, Italy) equipped with a 7 to 12 MHz linear transducer and the automated software guided technique radiofrequency—Quality Intima Media Thickness in real-time (QIMT, Esaote, Maastricht, Holland)—was used [13]. Patients from Lugo were assessed using high-resolution B-mode ultrasound, Hewlett Packard SONOS 5500, with a 10-MHz linear transducer as previously reported [14]. cIMT was measured at the far wall of the right and left common carotid arteries, 10 mm from the carotid bifurcation, over the proximal 15 mm-long segment. cIMT was determined as the average of three measurements in each common carotid artery. The final cIMT was the largest average cIMT (left or right). Agreement between the two US methods in patients with RA was previously reported [15]. Two experts with extensive experience and close collaboration in the assessment of cIMT in RA from Santander (AC) and Lugo (CGJ) performed the studies.

### Statistical analysis

Results are displayed as mean and standard deviation (SD). The association between the genotypes of the *ZC3HC1* rs11556924 polymorphism and cIMT values was tested using the Mann–Whitney test to compare between two groups. Comparisons of means was adjusted for sex, age at the time of the US study, follow-up time and traditional CV risk factors (hypertension, diabetes mellitus, dyslipidemia, obesity, and smoking habit) as potential confounders using analysis of covariance (ANCOVA).

Statistical significance was defined as *P* ≤0.05, and all analyses were performed using STATA statistical software 12/SE (Stata Corp., College Station, TX, USA).

## Results

The genotyping success was 99% and *ZC3HC1* rs11556924 genotype distribution was in Hardy-Weinberg equilibrium.

Results of the comparison between the different genotypes of *ZC3HC1* rs11556924 polymorphism according to cIMT are shown in Table 2. RA patients carrying the TT genotype had significantly higher cIMT values than those homozygous for the CC genotype (mean ± SD: 0.76 ± 0.18 mm and mean ± SD: 0.71 ± 0.16 mm, respectively; *P* = 0.03) while patients carrying the CT genotype had intermediate cIMT values (mean ± SD: 0.73 ± 0.17 mm) (CT versus CC *P* = 0.16).

**Table 2:** **Association between *ZC3HC1 *** **rs11556924 genotypes and carotid intima-media thickness (cIMT) in RA patients**

	**cIMT mm mean ± SD (n)**	***P***
*ZC3HC1* rs11556924		
Genotype distribution		
CC	0.71 ± 0.16 (175)	Ref.
CT	0.73 ± 0.17 (244)	0.16
TT	0.76 ± 0.18 (83)	0.03

RA, rheumatoid arthritis; SD, standard deviation.

Since sex, age at the time of US study, follow-up time and traditional CV risk factors (hypertension, diabetes mellitus, dyslipidemia, obesity, and smoking habit) may act as potential confounders of the results derived from the US assessment, adjustment for these potential confounders was performed using an ANCOVA model. Interestingly, even after adjusting for potential confounders, patients carrying the TT genotype had significantly higher cIMT values than those carrying the CC genotype (*P* = 0.04) showing that the effect conferred by the *ZC3HC1* rs11556924 polymorphism is independent of the traditional CV risk factors.

## Discussion

Cardiovascular disease is the most common cause of premature mortality in patients with RA, being a consequence of accelerated atherosclerosis [1]. The augmented CV mortality observed in this pathology is the result of a compound effect mediated by traditional CV risk factors, chronic inflammation [3] and the genetic component [4]. Since genes have been associated with an increased risk of CV disease in RA [4], in recent years several studies have been focused on the search for genetic markers that may improve the identification of RA patients at risk of experiencing CV events. Interestingly, cIMT has been proposed to be a good predictor of CV events in low and intermediate CV risk groups of non-rheumatic individuals and also in RA patients [8]. Also, a recent study has supported the use of carotid US in the assessment of the CV risk of RA patients [13].

A large-scale study has disclosed several loci associated with CAD [9]. Interestingly, in this study the *ZC3HC1* rs11556924 polymorphism was associated with CAD in non-rheumatic Caucasian individuals [9]. Therefore, in spite of having a moderate effect on CAD as compared to other genetic variants described by Schunkert *et al.,* such as polymorphisms in *CDKN2A, SORT1, LDLR, MRPS6* and *MIA3*[9], we assessed this time the *ZC3HC1* rs11556924 polymorphism because we had already analyzed the potential role of some others. In this regard, we previously showed that the rs599839 polymorphism located in the lp13.3 genomic region (*SORT1*) was associated with endothelial dysfunction in RA [16]. In addition, we reported an association between *MIA3* rs17465637 A allele with the risk of CV events in RA patients with dyslipidemia [17]. The assessment of the potential influence of other polymorphisms in the risk of CV disease in RA, such as *CDKN2A* and *CDKN2B*, *LDLR, MRPS6, PPAP2B and ADAMST7*, among others, is still underway.

ZC3HC1 (zinc finger, C3HC-type containing 1), also called NIPA (nuclear interaction partner of ALK), is a mammalian F-box-like protein [18] that monitors the timing of mitotic entry and in complex with constitutively active oncogenic proteins contributes to the development of carcinogenesis [19]. Since it has been shown that mediators of angiogenesis may play an important role in the regulation of endothelial integrity and inflammation [20], it is possible that changes in the stability and functional properties of ZC3HC1 protein may play a role in the endothelial dysfunction and, in the long run, in the development of atherosclerosis.

Since the incidence of CV disease is increased in patients with RA, we assessed for the first time the potential association between this polymorphism (as a marker of CV disease) and subclinical atherosclerosis in RA. Our results show that RA patients carrying the TT genotype have significantly higher cIMT values than those RA patients carrying the CC genotype, even after adjusting for potential confounders, supporting the evidence that the genetic component plays a relevant role in the development of CV disease in RA [4]. These results showing an association with the TT genotype apparently seem to be in contradiction with the data described by Schunkert *et al.* that reported an association of the C allele with the risk of CAD [9]. However, it is important to highlight that the populations analyzed in our study and in the study by Schunkert *et al.* are different. In this regard, Schunkert *et al.* performed their study in non-rheumatic Caucasian individuals while we assessed Spanish RA patients with a chronic inflammatory disease. Therefore, while in patients with RA the presence of a chronic inflammatory burden (among other factors) may lead to accelerated atherosclerosis, it may not be the case for the development of atherosclerosis in healthy individuals. Thus, differences in the mechanisms implicated in the pathogenesis of atherosclerosis may account for these results. However, further independent replication studies are required to confirm our results in patients with RA.

## Conclusion

Our results indicate that the *ZC3HC1* rs11556924 polymorphism is associated with subclinical atherosclerosis in RA.

## Abbreviations

ACR: American College of Rheumatology; ANCOVA: Analysis of covariance; CAD: Coronary artery disease; cIMT: Carotid intima-media thickness; CV: Cardiovascular; PCR: Polymerase chain reaction; RA: Rheumatoid arthritis; SD: Standard deviation; SNP: Single-nucleotide polymorphism; US: Ultrasonography; ZC3HC1: Xinc finger C3HC-type containing 1; αCCP: Anti-cyclic citrullinated protein/peptide antibodies.

## Competing interest

The authors declare that they have no competing interests.

## Authors’ contributions

RLM, FG and MGB carried out genotyping, participated in the design of the study, data analysis and drafted the manuscript. AC and CGJ performed the carotid US examination and were involved in the acquisition, interpretation of data and coordination and helped to draft the manuscript. JL carried out analysis and interpretation of the data. JAMF, JRG, RB and SC were involved in the acquisition and interpretation of data and in revising the manuscript critically for important intellectual content. JM and MAG-G have made substantial contributions to the conception and design of the study, acquisition of data, coordination and helped to draft the manuscript and have given final approval of the version to be published. All authors have read and approved the final manuscript.

## Authors’ information

Drs. Miguel A. González-Gay and Javier Martín shared senior authorship in this study.
